# A deep learning model for the interpretable identification of pulmonary thromboembolism from computed tomography pulmonary angiography

**DOI:** 10.3389/fcvm.2026.1864916

**Published:** 2026-07-24

**Authors:** Yemei Li, Sunyu Chen, Guangkun Chen, Baosheng Ren, Shibing Hu, Min Zong, Zhongzhi Jia, Tongqing Xue, Ganzhu Feng, Dancen Li

**Affiliations:** 1 Department of Pulmonary and Critical Care Medicine, The Third Affiliated Hospital of Nanjing Medical University (Changzhou Second People's Hospital), Changzhou, China; 2 Department of Interventional and Vascular Surgery, The Third Affiliated Hospital of Nanjing Medical University (Changzhou Second People's Hospital), Changzhou, China; 3 Department of Oncology and Vascular Intervention, Gaochun People's Hospital, Nanjing, China; 4 Department of Radiology, The First Affiliated Hospital With Nanjing Medical University (Jiangsu Province Hospital), Nanjing, China; 5 Department of Interventional Radiology, Huaian Hospital of Huai'an City (Huaian Cancer Hospital), Huai'an, China; 6 Department of Pulmonary and Critical Care Medicine, The Second Affiliated Hospital of Nanjing Medical University, Nanjing, Jiangsu, China; 7 Department of Neurosurgery, The Third Affiliated Hospital of Nanjing Medical University (Changzhou Second People's Hospital), Changzhou, China

**Keywords:** computed tomography pulmonary angiography, deep learning, interpretability, pulmonary thromboembolism, YOLOv11

## Abstract

**Objective:**

The rapid identification of pulmonary thromboembolism (PTE) on computed tomography pulmonary angiography (CTPA) is vital but labor-intensive, often leading to diagnostic delays. We aimed to construct and evaluate a YOLOv11 object detection algorithm capable of automatically highlighting intraluminal filling defects to expedite emergency radiological workflows.

**Methods:**

A retrospective analysis was conducted on CTPA scans from multiple centers. The dataset was divided into a primary internal cohort (*n* = 1,368) for model derivation and testing, alongside an independent external cohort (*n* = 98) to assess generalizability. The diagnostic efficacy of the YOLOv11 architecture was quantified using the area under the receiver operating characteristic curve (AUC), sensitivity, and specificity. Additionally, gradient-weighted class activation mapping (Grad-CAM) was applied to map the spatial distribution of the model's focus, ensuring clinical transparency.

**Results:**

During internal testing, the proposed framework yielded an AUC of 0.777 [95% confidence interval (CI): 0.765–0.788], corresponding to a sensitivity of 74.53% and a specificity of 64.26%. When applied to the external cohort, the algorithm's discriminative ability remained consistent with an AUC of 0.778 (95% CI: 0.749–0.806). Notably, the external sensitivity reached 86.75% (specificity: 54.46%). Visual assessments via Grad-CAM saliency maps confirmed that the model accurately localized embolic occlusions within the complex pulmonary arterial tree.

**Conclusion:**

Utilizing the YOLOv11 architecture for automated CTPA analysis yields a highly sensitive and visually interpretable screening mechanism. This artificial intelligence-assisted approach holds substantial promise for reducing missed diagnoses and accelerating patient triage in acute clinical settings.

## 1 Introduction

The swift and accurate diagnosis of acute pulmonary thromboembolism (PTE) remains a formidable challenge in cardiovascular emergency medicine (1). While computed tomography pulmonary angiography (CTPA) serves as the definitive reference standard, a modern protocol generates massive datasets that are exceptionally time-consuming to review manually (2). This high-volume workload increases the vulnerability to diagnostic oversights, particularly within the tortuous distal segmental and subsegmental pulmonary branches (3). Distinguishing true embolic occlusions from flow artifacts, respiratory motion, or complex vascular bifurcations often leads to fatigue-induced errors and notable variations in inter-reader reliability (4, 5), highlighting an acute necessity for intelligent computational aids to standardize the screening process (6, 7).

Previous systematic reviews have consolidated the evidence for deep learning in PTE detection. Meanwhile, novel segmentation-driven approaches and dual-pronged frameworks combining detection and segmentation have advanced the field (8–10). Our work extends these efforts by focusing on a single-stage detector (YOLOv11) with explicit interpretability via Grad-CAM, aiming for rapid emergency triage.

Recent advancements in computer vision have positioned single-stage object detection networks as viable second-reader systems to mitigate these diagnostic bottlenecks. However, balancing real-time computational efficiency with the granular spatial precision required to detect subtle intraluminal filling defects is a persistent hurdle. The YOLOv11 architecture emerges as a highly suitable candidate to address this specific gap (11). Our research group has previously evaluated the performance of YOLOv8 for medical image detection tasks (12), establishing its feasibility for identifying focal abnormalities. Building upon those findings, we selected the more recent YOLOv11 architecture for this multicenter clinical validation, capitalizing on its improved parameter efficiency and the C2PSA attention mechanism. Compared with two-stage detectors such as Faster R-CNN, single-stage YOLO models offer a favorable trade-off between inference speed and localization accuracy, which is critical for processing hundreds of CTPA slices per patient in acute triage scenarios. By integrating advanced structural optimizations—such as the C2PSA mechanism—this framework excels at capturing intricate spatial dependencies (13). This makes it uniquely equipped to navigate the complex branching patterns of the pulmonary arterial tree without compromising inference speed.

While deep learning algorithms offer robust predictive capabilities, their inherent “black-box” nature frequently impedes clinical trust and real-world adoption. Therefore, this investigation prioritized both clinical utility and diagnostic transparency. The primary objective of this study was to construct and validate a YOLOv11-driven diagnostic pipeline for automated PTE detection using multicenter cohorts. Crucially, we utilized gradient-weighted class activation mapping (Grad-CAM) to visually substantiate the network's reasoning (14). By directly bridging algorithmic outputs with recognizable pathological anatomy, this approach aims to deliver a reliable and explainable decision-support mechanism for frontline practitioners.

## 2 Materials and methods

### 2.1 Patient selection and ethical approval

Approval for this retrospective, multi-institutional analysis was granted by the respective ethical review boards of the three participating centers. Given the purely retrospective nature of the image analysis, patients informed consent requirements were waived.

We screened consecutive adult patients (≥18 years of age) evaluated suspected acute pulmonary thromboembolism between January 2013 and October 2025. Patients were considered eligible if they had undergone a dedicated computed CTPA examination. To ensure the reliability of the deep learning model, we rigorously excluded cases exhibiting: (1) significant imaging artifacts or inadequate contrast opacification; (2) missing baseline clinical records; or (3) confounding thoracic pathologies (e.g., advanced malignancies, massive consolidation, or prior anatomical resections) that could severely distort the natural pulmonary vascular architecture.

The primary internal dataset comprised individuals treated at Changzhou Second People's Hospital and Jiangsu Province People's Hospital. To mitigate baseline demographic imbalances, a 1:1 propensity score matching (PSM) protocol based on age and gender was executed, yielding a balanced cohort of 1,368 patients (684 PTE-positive and 684 negative controls). This internal cohort was subsequently randomized into a training subset (70%) and an internal validation subset (30%). For independent verification, an external validation cohort (*n* = 98) was established using unseen data from the other three hospitals, adhering to the identical 1:1 PSM criteria.

### 2.2 Image acquisition and ground truth establishment

All pulmonary angiographic studies were acquired utilizing 64-slice multi-detector row scanners (Somatom Definition Flash, Siemens Healthineers; or Core 128, Philips Healthcare). Acquisition parameters were standardized to a tube voltage of 120 kVp with automated current modulation. We extracted thin-section axial reconstructions (1.0 mm or 1.5 mm slice thickness) with an in-plane matrix of 640 × 640 pixels.

Prior to algorithmic exposure, all imaging data were strictly anonymized. A panel of three senior thoracic radiologists, each possessing over a decade of subspecialty experience, conducted a rigorous quality control phase. Scans demonstrating excessive breathing motion or suboptimal vascular enhancement—strictly defined as less than 250 Hounsfield units (HU) within the main pulmonary artery—were discarded.

For the ground truth annotation, three board-certified radiologists participated. Each CTPA scan was independently reviewed by two radiologists, who localized confirmed intraluminal filling defects and drew bounding boxes using LabelImg. Any inter-reader discrepancies were adjudicated through a joint review session with the third radiologist, and a unanimous final decision was reached prior to annotation. A final cross-verification step was implemented to ensure that subtle peripheral clots were not omitted.

Negative samples (CT slices without any embolic filling defect) were intentionally retained in the training set to teach the model to suppress false positives caused by flow artifacts or complex vascular bifurcations. For ambiguous cases at vessel bifurcations, the bounding box was drawn to cover the entire visible hypodense region within the parent branch; border artifacts at the edge of the field of view were excluded by consensus. Any inter-reader discrepancies were resolved via joint review to reach a final diagnostic consensus prior to bounding box annotation, ensuring the highest possible quality for the ground truth.

### 2.3 YOLOv11 network configuration and optimization

The computational pipeline was anchored by the YOLOv11 object detection framework (Ultralytics), deployed within a Python 3.12 and PyTorch environment. Hardware acceleration was provided by an NVIDIA GeForce RTX 3090 graphical processing unit (24 GB VRAM). To bypass cold-start inefficiencies, the single-stage detector was initialized with pre-trained weights from the Common Objects in Context (COCO) dataset.

We specifically leveraged the YOLOv11 architecture for its advanced structural integrations, which are highly suited for pulmonary vascular imaging. The C3k2 module was utilized to optimize gradient flow, while the C2PSA (Position-Sensitive Attention) mechanism was critical for spatial contextualization. This attention mechanism is particularly adept at differentiating true intraluminal clots from adjacent high-contrast anatomical boundaries within the tortuous pulmonary arterial tree. To mitigate model overfitting and improve anatomical generalization, dynamic data augmentation strategies—including mosaic composition, stochastic scaling, and horizontal flipping—were applied to the CTPA arrays. Network optimization was governed by a multitask loss function designed to simultaneously penalize classification errors and bounding box regression inaccuracies. The key hyperparameters used for YOLOv11 training are summarized in Table 1. All other parameters followed the default settings of the Ultralytics YOLOv11 implementation.

**Table 1:** Training hyperparameters for the YOLOv11 model.

Hyperparameter	Value
Optimizer	Adam
Initial learning rate	5e^-3^
Learning rate schedule	Cosine decay to 0.000298 at epoch 50
Number of epochs	50
Batch size	64
Input image size	640 × 640 pixels
Confidence threshold	0.5
NMS IoU threshold	0.25
Data augmentation	Horizontal and vertical flipping

### 2.4 Diagnostic performance metrics and patient-level aggregation

**Figure 1:**
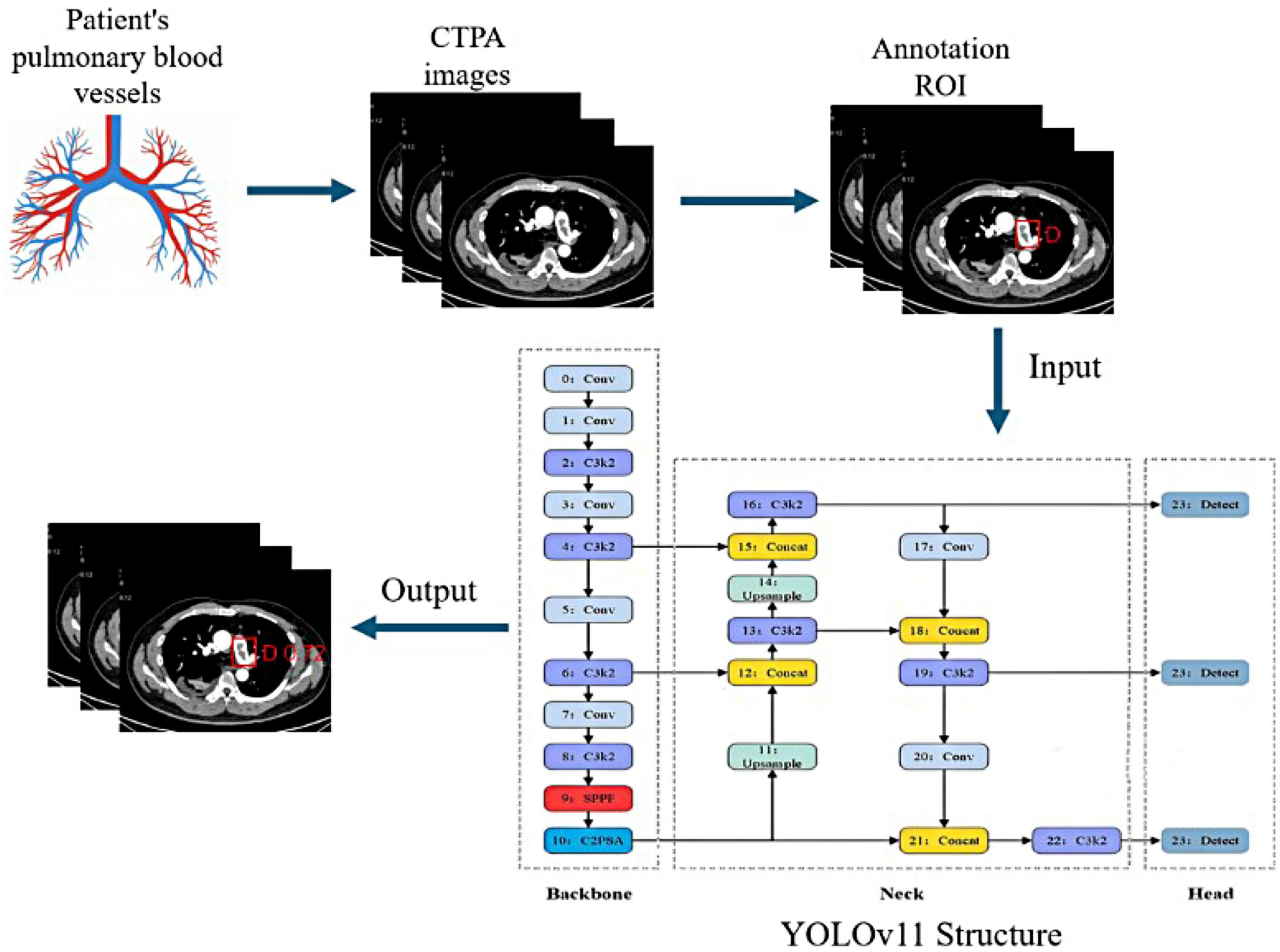
Overview of the image annotation scope, YOLOv11 model architecture, and training workflow. CTPA, computed tomography pulmonary angiography. ROI, region of interest.

A comprehensive overview of our analytical pipeline—encompassing the initial image annotation scope, the YOLOv11 structural configuration, and the ultimate output generation—is visually summarized in Figure 1. To translate the resulting slice-level algorithmic outputs into actionable, patient-level clinical diagnoses, we engineered a specific aggregation protocol. The YOLOv11 network assigned a continuous probability score (0–1) to each thin-layer slice. For any given CTPA study, the global probability of a PTE diagnosis was calculated as the mean class probability across all evaluated axial images. This mean-probability aggregation method was chosen to avoid over-weighting isolated false positives on individual slices (e.g., due to transient artifacts), thereby providing a robust patient-level decision.

The discriminative capacity of the model was quantitatively assessed using receiver operating characteristic (ROC) curves. The area under the curve (AUC), accompanied by 95% confidence intervals (CIs), served as the primary global performance indicator. Furthermore, operating thresholds were statistically optimized to calculate sensitivity and specificity, with a strategic clinical emphasis on maximizing sensitivity to avert catastrophic false-negative triages in acute emergency settings.

### 2.5 Algorithmic interpretability via saliency mapping

To pierce the “black-box” nature of deep learning networks and foster diagnostic trust among interpreting physicians, we integrated Grad-CAM into the output pipeline. This technique generates topological heatmaps superimposed directly onto the original CTPA slices. By visually highlighting the specific pixel clusters that drove the model's positive predictions, we could continuously verify that the algorithm genuinely isolating embolic filling defects within the vascular lumen, rather than anchoring its decisions on peripheral parenchymal noise or imaging artifacts.

### 2.6 Statistical analysis

Statistical computing and graphical visualizations were executed via IBM SPSS Statistics (Version 26.0) and GraphPad Prism (Version 9.0). The propensity scores matching procedure utilized a rigorous nearest-neighbor matching algorithm without replacement to ensure strict demographic parity between the PTE and control cohorts.

Normality of continuous variables was assessed using standard distribution testing; these data were subsequently reported as means with standard deviations or medians with interquartile ranges and compared using independent samples t-tests or Mann–Whitney U tests, respectively. Categorical distributions were reported as frequencies and proportions, evaluated via Pearson's Chi-square or Fisher's exact tests as dictated by expected cell counts. Statistical significance was defined as *a priori* at a two-tailed alpha level of less than 0.05.

## 3 Results

### 3.1 Characteristics of the analytic cohorts

**Figure 2:**
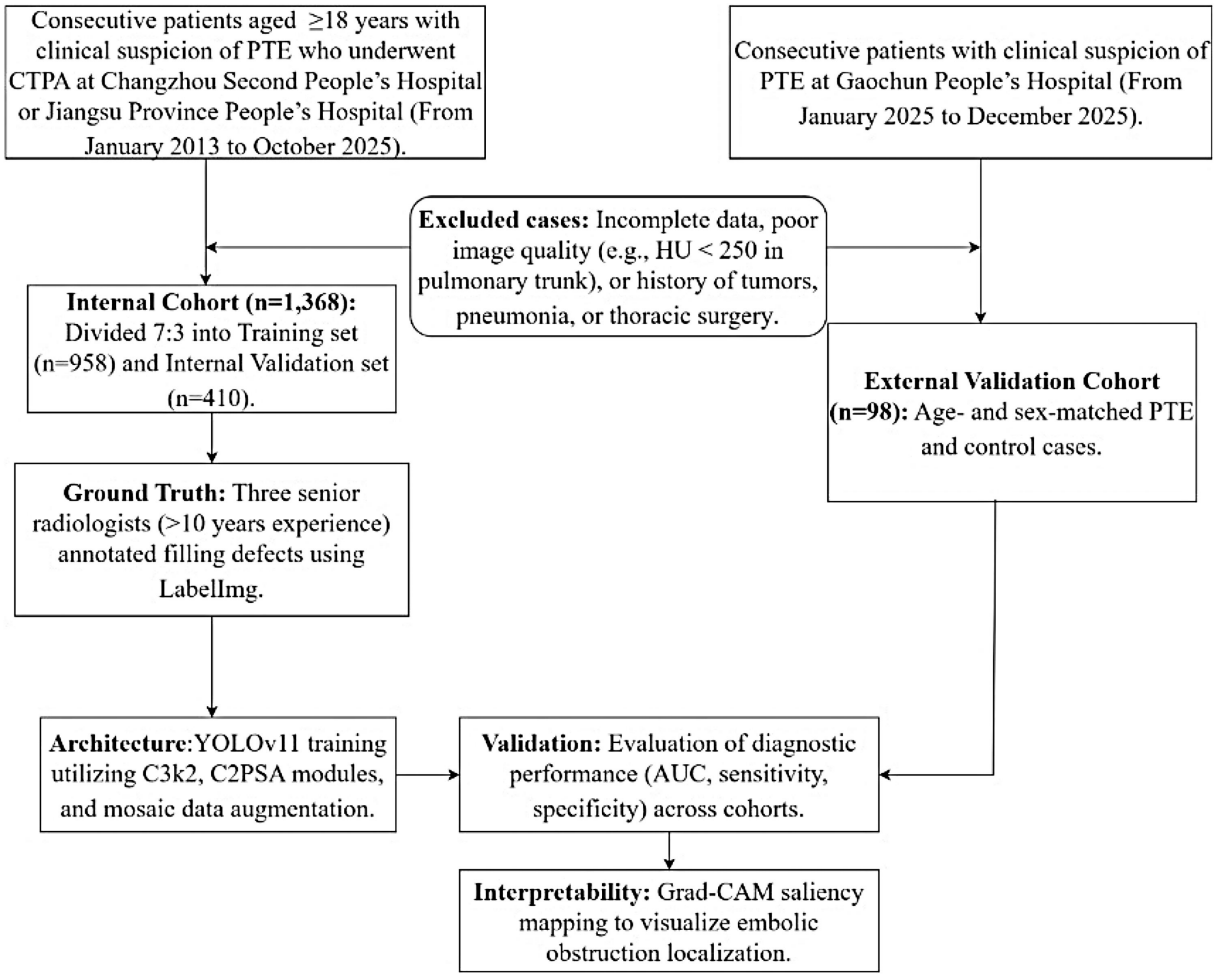
Flowchart for the identification of PTE via CTPA using YOLOv11.

Following the rigorous application of inclusion and exclusion criteria outlined in the patient selection flowchart (Figure 2), the final analytic sample comprised 1,466 consecutive patient studies evaluated between January 2013 and October 2025. The primary derivation and internal testing dataset encompassed 1,368 individuals. PSM effectively balanced this internal pool, resulting in 684 patients with radiologically confirmed PTE and an equal number of patent-vessel controls, ensuring demographic parity across both groups (Table 2). This primary dataset was partitioned into a 70% training subset and a 30% internal testing subset. Concurrently, the independent external validation group compiled 98 PSM-balanced cases (49 confirmed PTE, 49 controls) to rigorously test the algorithm's generalizability across different clinical sites. A comprehensive breakdown of the dataset is presented in Table 3.

**Table 2:** Baseline characteristics of the study population.

Characteristics	Internal Cohort (*n* = 1,368)		External Cohort (*n* = 98)	
	PTE (*n* = 684)	Control (*n* = 684)	PTE (*n* = 49)	Control (*n* = 49)
Age (year)	65.3 ± 14.4	69.9 ± 14.7	73.0±13.7	73.4±14.0
Sex (Male/Female)	369/315	369/315	23/26	23/26

**Table 3:** Dataset characteristics.

Parameter	Internal Cohort	External Cohort
Total CTPA scans	1,368	98
Total slices	8,946	625
Total annotated emboli	5,732	412
Distribution of emboli		
Main pulmonary artery	286 (5.0%)	18 (4.4%)
Lobar	974 (17.0%)	71 (17.2%)
Segmental	2,521 (44.0%)	187 (45.4%)
Subsegmental	1,951 (34.0%)	136 (33.0%)

### 3.2 Algorithmic efficacy in embolus detection

**Figure 3:**
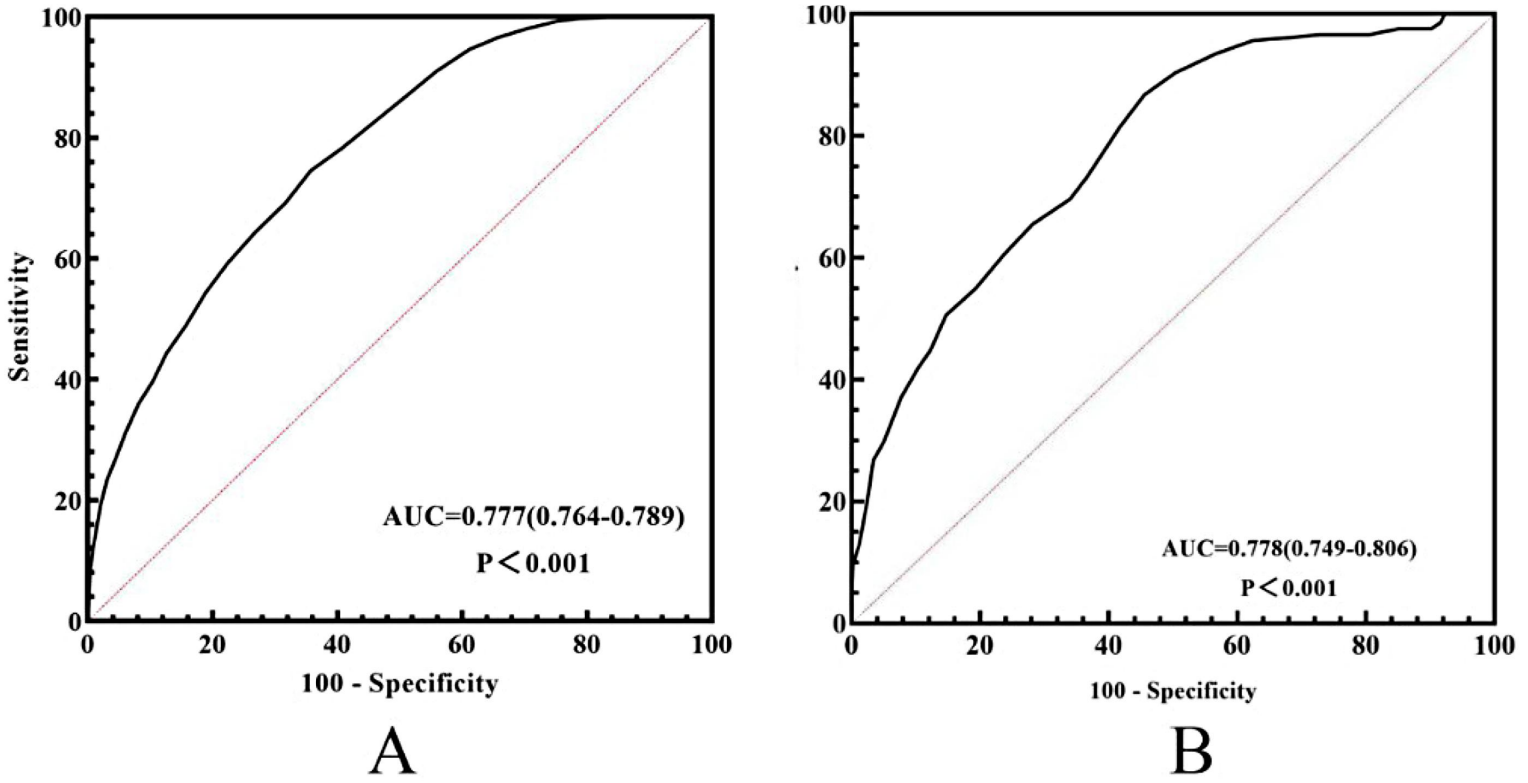
Receiver operating characteristic curve for the internal validation cohort **(A)** and external validation cohort **(B).**

The diagnostic and localization performance of the YOLOv11-based framework was evaluated using both classification and object detection metrics. For patient-level diagnosis, the model yielded an AUC of 0.777 (95% CI: 0.765–0.788) (Figure 3A).

In addition to classification-level metrics, standard object detection metrics were computed on the internal validation set to provide algorithmic transparency. At the best validation epoch, the model achieved a Precision of 0.68, Recall of 0.65, F1-score of 0.67, mean Average Precision at IoU threshold 0.5 (mAP@0.5) of 0.67, and mAP@0.5:0.95 of 0.25. IoU statistics were not separately logged; however, the mAP values implicitly reflect the overall localization performance. A discussion of the discrepancy between clinical triage success and strict bounding box regression is provided in the Discussion section.

Upon transitioning to the completely unseen external cohort, the overall discriminative strength remained remarkably robust, producing an AUC of 0.778 (95% CI: 0.749–0.806), as illustrated in the corresponding ROC curve (Figure 3B). Most notably, at the ≤ 0.69 operating threshold, the model's external sensitivity surged to 86.75% (with a corresponding specificity of 54.46%). This distinctively high sensitivity underscores the model's aggressive profiling for potential embolic events—a highly desirable characteristic for frontline emergency screening tools where minimizing catastrophic missed diagnoses takes clinical precedence over false-positive alerts. Of note, the internal and external cohorts used slightly different probability thresholds (≤0.67 vs. ≤ 0.69) optimized for maximum sensitivity in the respective populations, which contributed to the higher external recall.

Per-box IoU distributions were not separately logged; however, mAP values, which are derived from IoU thresholds, were recorded and reported.

### 3.3 Visual substantiation via saliency heatmaps

**Figure 4:**
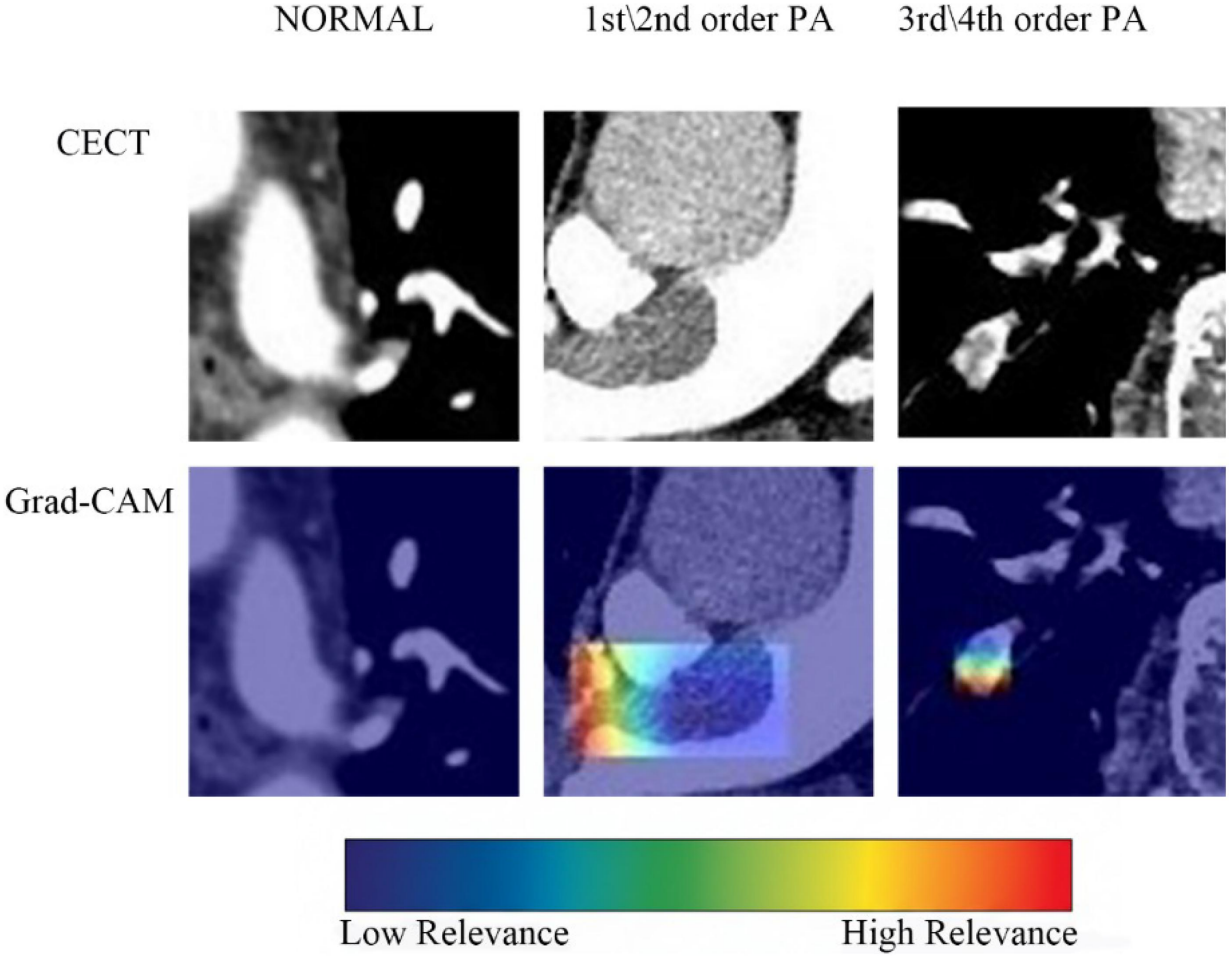
Visualization results of Grad-CAM across different grades. CECT, contrast-enhanced CT; PA, pulmonary artery; Grad-CAM, gradient-weighted class activation mapping.

Algorithmic transparency was evaluated through the generation of Grad-CAM saliency maps on the CTPA arrays. Visual inspection of these topological overlays confirmed that the YOLOv11 network was actively deriving its predictions from pertinent intravascular pathology, rather than extraneous imaging noise. The highest activation signals consistently mapped to the precise anatomical coordinates of intraluminal filling defects. Crucially, the model maintained this spatial accuracy even when navigating distal, higher-order pulmonary branches, successfully isolating occlusive clots amidst complex vascular bifurcations (Figure 4).

## 4 Discussion

This investigation successfully engineered and validated an automated computational pipeline leveraging the YOLOv11 architecture for the rapid identification of acute PTE on CTPA datasets. Our proposed network exhibited consistent discriminative capacity, yielding an internal validation AUC of 0.777 (sensitivity: 74.53%, specificity: 64.26%) and maintaining a robust AUC of 0.778 in the external cohort, where it notably achieved a heightened sensitivity of 86.75% for localizing intraluminal clots.

While the overall AUC of 0.777 may appear moderate compared to benchmarks in other domains, several factors contextualize this result. First, our external validation demonstrated an exceptionally high sensitivity of 86.75%, which is clinically paramount in acute triage to minimize catastrophic misses. Second, as shown in Table 2, a substantial proportion (34.0%) of our annotated emboli are subsegmental, which are notoriously difficult to detect due to their small caliber and complex bifurcation morphology. For such lesions, a model that successfully flags the embolus to the radiologist may still receive a low IoU score due to minor bounding-box deviations, thus penalizing the mAP and, by extension, the classification performance derived from detection confidences. Therefore, we argue that sensitivity and AUC, when interpreted in the context of the clinical workflow, provide a more pragmatic measure of the model's utility than strict localization metrics alone.

The prompt and accurate identification of acute PTE is imperative for patient prognosis, yet initial clinical evaluations are frequently confounded by atypical presentations (1). Furthermore, the meticulous interrogation of modern CTPA studies—often comprising hundreds of sub-millimeter transaxial images—imposes a substantial cognitive burden on radiologists, precipitating diagnostic delays and interpretation variability (15). The deployment of deep learning-driven object detection frameworks offers a pragmatic solution to alleviate these workflow bottlenecks and standardize emergency radiological assessments (16). This aligns with the growing body of evidence from systematic reviews and recent methodological innovations (8–10), which collectively underscore the potential of AI-assisted PTE detection.

Subsegmental emboli, which accounted for 34% of our annotations, often occupied fewer than 32 × 32 pixels at 640 × 640 resolution; this small target size partly explains the relatively low mAP@0.5:0.95 of 0.25. Although the overall AUC reflects a moderate global accuracy, the algorithm's exceptional sensitivity (86.75%) during external validation warrants particular emphasis. Within high-stakes emergency environments, preliminary screening mechanisms must prioritize sensitivity to safeguard against catastrophic false-negative triages (17). The primary architectural intent of our YOLOv11 deployment is to cast a wide safety net, aggressively flagging candidate emboli to ensure that subtle or distal occlusions are brought to the immediate attention of the reviewing radiologist.

Admittedly, this aggressive detection threshold resulted in a diminished specificity within the external dataset. Nevertheless, the parity of the AUC metrics between the internal and external cohorts (0.777 vs. 0.778) highlights the structural resilience of the YOLOv11 model. This metric stability confirms that the network has genuinely acquired the morphological features of intravascular thrombi, rather than merely memorizing institutional imaging artifacts or scanner-specific baseline variations (12).

The integration of Grad-CAM saliency mapping fundamentally addresses the “black-box” dilemma that historically impedes the clinical acceptance of artificial intelligence. By visually projecting the network's activation zones directly onto the CTPA slices, we verified its strict adherence to relevant pulmonary vascular anatomy. This visual transparency empowers interpreting physicians to instantaneously corroborate AI-generated alerts against true pathological features (18), thereby bolstering diagnostic confidence and mitigating visual fatigue during exhaustive slice-by-slice reviews.

From a translational perspective, the inherent computational economy of this framework facilitates seamless clinical integration (19). Operating as a single-stage detector, the YOLOv11 architecture processes volumetric data with near-real-time velocity, rendering it an ideal candidate for background deployment within hospital Picture Archiving and Communication Systems (PACS) to trigger automated, pre-read critical alerts (20).

Several limitations must be acknowledged. First, the modest sample size of the external validation cohort may restrict the precision of the generalized performance metrics. Second, the algorithm's propensity for lower specificity inherently generates a higher volume of false-positive flags. Third, the current iteration operates exclusively on imaging pixel data, omitting critical clinical covariates such as serum D-dimer concentrations and patient comorbidities, which mandate future multimodal investigations. Fourth, the present study did not include direct head-to-head comparisons of YOLOv11 with other detection architectures (e.g., YOLOv8, Faster R-CNN). Although our prior work had validated YOLOv8 for medical image detection, the absence of simultaneous benchmarking limits the ability to definitively claim algorithmic superiority. We have noted this as a limitation and plan to perform a systematic multi-architecture comparison in a dedicated follow-up study. Delving into the model's error profile, the observed false positives predominantly stem from inherent CTPA diagnostic pitfalls, such as the misclassification of unenhanced pulmonary veins, respiratory motion artifacts, or transient mucus plugs mimicking vascular defects (21). Subsequent research will aim to integrate multi-task learning paradigms to refine this specificity and potentially stratify PTE severity. Furthermore, expansive multicenter trials incorporating diverse patient demographics and heterogeneous imaging protocols are imperative to validate these findings prior to routine clinical implementation (22). Finally, the current framework processes each axial slice independently. While this 2D approach has already achieved clinically useful sensitivity, we acknowledge that incorporating inter-slice context could further improve the detection of small or obliquely oriented emboli. However, transitioning to full 3D networks is not trivial, as it would require prohibitively expensive 3D bounding box annotations and substantially higher computational resources for our 1,466-patient dataset. Future work will therefore explore 2.5D approaches or lightweight 3D convolutional networks to capture volumetric features while maintaining clinical and computational feasibility.

In conclusion, the proposed YOLOv11 framework delivers a robust and highly sensitive automated screening mechanism for the detection of PTE on thin-layer CTPA. Its aggressive candidate-flagging capability renders it an invaluable safeguard against missed emergency diagnoses. Complemented by the anatomical transparency of Grad-CAM visualizations, this approach presents a highly viable strategy to optimize radiological workflows and elevate the standard of acute pulmonary care.

## Data Availability

The data that support the findings of this study are not openly available due to patient privacy and confidentiality, but are available from the corresponding author upon reasonable request.

## Supplementary material

Supplementary Table S1 YOLOv11 Training Log (50 Epochs).

## References

[1] Creager MA Barnes GD Giri J Mukherjee D Jones WS Burnett AE. 2026 AHA/ACC/ACCP/ACEP/CHEST/SCAI/SHM/SIR/SVM/SVN guideline for the evaluation and management of acute pulmonary embolism in adults: a report of the American College of Cardiology/American Heart Association Joint Committee on Clinical Practice Guidelines. Circulation. (2026) 153:e977–1051. 10.1161/CIR.0000000000001415 41712677

[2] de Jong CMM Kroft LJM Van Mens TE Huisman MV Stöger JL Klok FA. Modern imaging of acute pulmonary embolism. Thromb Res. (2024) 238:105–16. 10.1016/j.thromres.2024.04.016 38703584

[3] Zuin M Bikdeli B Ballard-Hernandez J Barco S Battinelli EM Giannakoulas G. International clinical practice guideline recommendations for acute pulmonary embolism: harmony, dissonance, and silence. J Am Coll Cardiol. (2024) 84(16):1561–77. 10.1016/j.jacc.2024.07.044 39384264

[4] Chen D Bhaskar SMM. Pulmonary embolism in acute ischaemic stroke: evolving evidence, diagnostic challenges, and a novel thromboinflammatory axis hypothesis. Int J Mol Sci. (2025) 26(14):6733. 10.3390/ijms26146733 40724982 PMC12295995

[5] Götzinger F Lauder L Sharp ASP Lang IM Rosenkranz S Konstantinides S. Interventional therapies for pulmonary embolism. Nat Rev Cardiol. (2023) 20(10):670–84. 10.1038/s41569-023-00876-0 37173409 PMC10180624

[6] Amin KD Weissler EH Ratliff W Sullivan AE Holder TA Bury C. Development and validation of a natural language processing model to identify low-risk pulmonary embolism in real time to facilitate safe outpatient management. Ann Emerg Med. (2024) 84(2):118–27. 10.1016/j.annemergmed.2024.01.036 38441514

[7] Huang S-C Kothari T Banerjee I Chute C Ball RL Borus N. PENet-a scalable deep-learning model for automated diagnosis of pulmonary embolism using volumetric CT imaging. NPJ Digit Med. (2020) 3:61. 10.1038/s41746-020-0266-y 32352039 PMC7181770

[8] Chu Y Luo G Zhou L Cao S Ma G Meng X. Deep learning-driven pulmonary artery and vein segmentation reveals demography-associated vasculature anatomical differences. Nat Commun. (2025) 16(1):2262. 10.1038/s41467-025-56505-6 40050617 PMC11885638

[9] Bushra F Chowdhury MEH Sarmun R Kabir S Said M Bassam Zoghoul S. Deep learning in computed tomography pulmonary angiography imaging: a dual-pronged approach for pulmonary embolism detection. Expert Syst Appl. (2024) 245:123029. 10.1016/j.eswa.2023.123029

[10] Soffer S Klang E Shimon O Barash Y Cahan N Greenspana H. Deep learning for pulmonary embolism detection on computed tomography pulmonary angiogram: a systematic review and meta-analysis. Sci Rep. (2021) 11(1):15814. 10.1038/s41598-021-95249-3 34349191 PMC8338977

[11] Ma H Zhao Q Zhang R Hao C Dong W Zhang X. YOLOv11-GSF: an optimized deep learning model for strawberry ripeness detection in agriculture. Front Plant Sci. (2025) 16:1584669. 10.3389/fpls.2025.1584669 40909907 PMC12405203

[12] Tang Z Huang Y Hu S Shen T Meng M Xue T. Deep learning application of YOLOv8 for aortic dissection screening using non-contrast computed tomography. Eur J Vasc Endovasc Surg. (2026) 71(4):687–95. 10.1016/j.ejvs.2025.08.054 40902929

[13] Sazak H Kotan M. Automated blood cell detection and classification in microscopic images using YOLOv11 and optimized weights. Diagnostics (Basel). (2024) 15(1):22. 10.3390/diagnostics15010022 39795550 PMC11719705

[14] Fayyaz AM Abdulkadir SJ Talpur N Al-Selwi SM Hassan SU Sumiea EH. Grad-CAM (gradient-weighted class activation mapping): a systematic literature review. Comput Biol Med. (2025) 198(Pt B):111200. 10.1016/j.compbiomed.2025.111200 41108904

[15] Choi Y. Leveraging GPT-4 as a proofreader: addressing the growing workload of radiologists. Radiological Society of North America. (2025) 314(1):e243859. 10.1148/radiol.243859 39873608

[16] Hu C Xia Y Zheng Z Cao M Zheng G Chen S. AI-based large-scale screening of gastric cancer from noncontrast CT imaging. Nat Med. (2025) 31(9):3011–9. 10.1038/s41591-025-03785-6 40555751 PMC12443630

[17] Hu Y Xiang Y Zhou Y-J He Y Lang D Yang S. AI-based diagnosis of acute aortic syndrome from noncontrast CT. Nat Med. (2025) 31(11):3832–44. 10.1038/s41591-025-03916-z 40835970 PMC12618251

[18] Cai JC Nakai H Kuanar S Froemming AT Bolan CW Kawashima A. Fully automated deep learning model to detect clinically significant prostate cancer at MRI. Radiology. (2024) 312(2):e232635. 10.1148/radiol.232635 39105640 PMC11366675

[19] Yu F Moehring A Banerjee O Salz T Agarwal N Rajpurkar P. Heterogeneity and predictors of the effects of AI assistance on radiologists. Nat Med. (2024) 30(3):837–49. 10.1038/s41591-024-02850-w 38504016 PMC10957478

[20] Swinburne NC Yadav V Kim J Choi YR Gutman DC Yang JT. Semisupervised training of a brain MRI tumor detection model using mined annotations. Radiology. (2022) 303(1):80–9. 10.1148/radiol.210817 35040676 PMC8962822

[21] Pu J Gezer NS Ren S Alpaydin AO Avci ER Risbano MG. Automated detection and segmentation of pulmonary embolisms on computed tomography pulmonary angiography (CTPA) using deep learning but without manual outlining. Med Image Anal. (2023) 89:102882. 10.1016/j.media.2023.102882 37482032 PMC10528048

[22] Sadegh-Zadeh S-A Sakha H Movahedi S Fasihi Harandi A Ghaffari S Javanshir E. Advancing prognostic precision in pulmonary embolism: a clinical and laboratory-based artificial intelligence approach for enhanced early mortality risk stratification. Comput Biol Med. (2023) 167:107696. 10.1016/j.compbiomed.2023.107696 37979394

