# Supplementary material for: A deep learning model for the interpretable identification of pulmonary thromboembolism from computed tomography pulmonary angiography
Source: Front Cardiovasc Med. 2026 Jul 24;13:1864916. doi: 10.3389/fcvm.2026.1864916 (PMC13447075; doi:10.3389/fcvm.2026.1864916)
Supplement: Supplementary file 1 [file Table1.pdf]

Table S1. YOLOv11 Training Log (50 Epochs)

epoch	time	precision	recall	mAP50	mAP50-95	F1-score
1	242.43	0.42	0.34	0.32	0.12	0.38
2	465.36	0.60	0.49	0.52	0.19	0.54
3	684.01	0.54	0.50	0.48	0.16	0.52
4	901.03	0.52	0.46	0.44	0.16	0.49
5	1117.67	0.60	0.53	0.53	0.19	0.56
6	1333.95	0.59	0.54	0.54	0.19	0.57
7	1550.28	0.61	0.55	0.57	0.20	0.58
8	1765.96	0.63	0.59	0.60	0.22	0.61
9	1981.90	0.63	0.59	0.59	0.21	0.61
10	2198.13	0.62	0.58	0.59	0.21	0.60
11	2413.67	0.63	0.57	0.60	0.22	0.60
12	2629.66	0.65	0.58	0.60	0.22	0.62
13	2844.85	0.66	0.61	0.63	0.23	0.63
14	3060.56	0.64	0.61	0.62	0.23	0.63
15	3276.54	0.63	0.59	0.61	0.21	0.61
16	3491.71	0.65	0.60	0.61	0.23	0.62
17	3707.68	0.66	0.60	0.61	0.24	0.63
18	3922.60	0.66	0.61	0.63	0.24	0.64
19	4138.01	0.65	0.61	0.62	0.23	0.63
20	4353.10	0.66	0.62	0.63	0.24	0.64
21	4568.48	0.68	0.62	0.64	0.24	0.65
22	4785.33	0.68	0.62	0.64	0.24	0.65

23	5000.38	0.67	0.64	0.66	0.24	0.66
24	5215.65	0.68	0.61	0.64	0.24	0.64
25	5431.16	0.66	0.64	0.65	0.24	0.65
26	5645.86	0.68	0.64	0.66	0.25	0.66
27	5862.34	0.68	0.65	0.66	0.25	0.66
28	6077.31	0.67	0.64	0.65	0.25	0.66
29	6292.07	0.68	0.65	0.66	0.24	0.66
30	6506.83	0.67	0.65	0.66	0.25	0.66
31	6721.38	0.68	0.65	0.66	0.25	0.66
32	6937.45	0.68	0.65	0.67	0.25	0.67
33	7152.10	0.68	0.65	0.67	0.25	0.67
34	7367.50	0.69	0.65	0.67	0.25	**0.67**
35	7582.82	0.69	0.65	0.67	0.26	0.67
36	7797.59	0.70	0.64	0.67	0.26	0.67
37	8012.77	0.69	0.64	0.66	0.25	0.67
38	8226.72	0.68	0.65	0.66	0.25	0.67
39	8441.82	0.69	0.64	0.65	0.25	0.66
40	8656.37	0.70	0.65	0.66	0.25	0.67
41	8907.24	0.70	0.64	0.66	0.26	0.67
42	9121.59	0.68	0.65	0.66	0.25	0.67
43	9335.68	0.67	0.66	0.66	0.25	0.66
44	9549.89	0.69	0.65	0.66	0.25	0.67

45	9764.89	0.68	0.65	0.65	0.25	0.66
46	9979.37	0.68	0.65	0.65	0.25	0.67
47	10194.00	0.67	0.66	0.66	0.25	0.66
48	10408.00	0.67	0.66	0.65	0.25	0.67
49	10621.90	0.67	0.66	0.66	0.25	0.67
50	10836.90	0.67	0.66	0.66	0.25	0.67
